# Supplementary material for: CD163 Monoclonal Antibody Modified Polymer Prodrug Nanoparticles for Targeting Tumor-Associated Macrophages (TAMs) to Enhance Anti-Tumor Effects
Source: Pharmaceutics. 2023 Apr 14;15(4):1241. doi: 10.3390/pharmaceutics15041241 (PMC10144748; doi:10.3390/pharmaceutics15041241)
Supplement: Supplementary file 1 [file pharmaceutics-15-01241-s001.zip › pharmaceutics-2270735-supplementary.pdf]

Supporting information for

CD163 Monoclonal Antibody Modified Polymer Prodrug Nanoparticles for Targeting Tumor-Associated Macrophages (TAMs) to Enhance Anti-Tumor Effects

Zun Yang [1], Haijiao Li [1], Wenrui Zhang [1], Mingzu Zhang [1], Jinlin He [1], Zepeng Yu [2], Xingwei Sun [3,*] and Peihong Ni [1,*]

[1] State and Local Joint Engineering Laboratory for Novel Functional Polymeric Materials, Jiangsu Key Laboratory of Advanced Functional Polymer Design and Application, Suzhou Key Laboratory of Macromolecular Design and Precision Synthesis, College of Chemistry, Chemical Engineering and Materials Science, Soochow University, Suzhou 215123, China
[2] Center for Medical Ultrasound, The Affiliated Suzhou Hospital of Nanjing Medical University, Suzhou 215001, China
[3] Intervention Department, The Second Affiliated Hospital of Soochow University, Suzhou 215004, China
* Correspondence: sdfeysxw@163.com (X.S.); phni@suda.edu.cn (P.N.); Tel.: +86-512-65882047 (P.N.)

Characterization

^{1}H NMR spectra were recorded on a 300 MHz spectrometer (INOVA-300, Varian), using $CDCl_3$ as the solvents and tetramethylsilane (TMS) as the internal standard. The number-average molecular weights ($\overline{M}_n$) and dispersity (*Đ*) of N_3-PEG-Br and N_3-PEG-*b*-PFBEMA were analyzed by gel permeation chromatography (GPC) instrument (HLC-8320, Tosoh) using polystyrene as the standard and THF as the eluent. The ultraviolet-visible (UV-vis) absorption spectra were conducted on a UV-vis spectrophotometer (UV-vis 1,900, Shimadzu), and fluorescence spectra were recorded on a fluorescence spectrophotometer (Cary Eclipse, Agilent). Fourier transform infrared (FT-IR) spectra (Vertex 70，Bruker TENSOR-27) using the KBr disk method. The self-assembly behavior of the polymer nanoparticles and morphological changes under different conditions were explored by dynamic light scattering (DLS, Zetasizer Nano ZS90, Malvern Instruments, UK) and transmission electron microscopy (TEM, Hitachi Limited HT7700).

Results

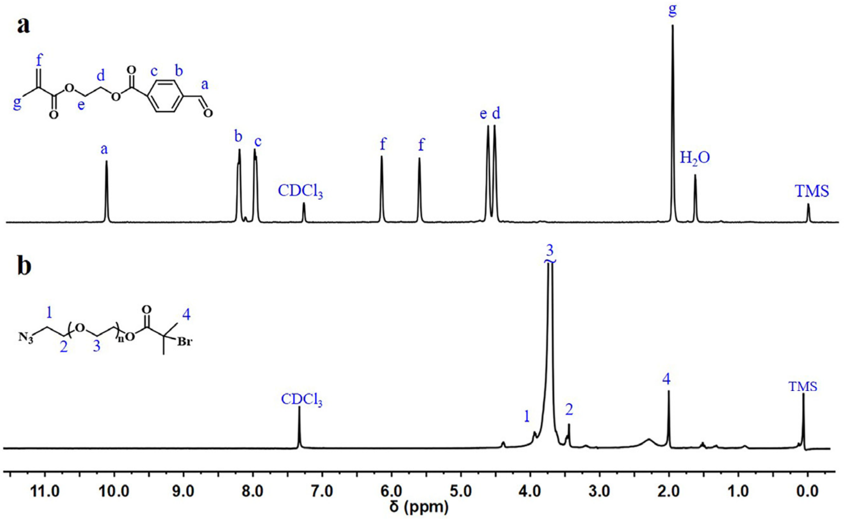

Figure S1. ^{1}H NMR spectra of (a) FBEMA monomer and (b) N_3-PEG-Br (solvent: $CDCl_3$).

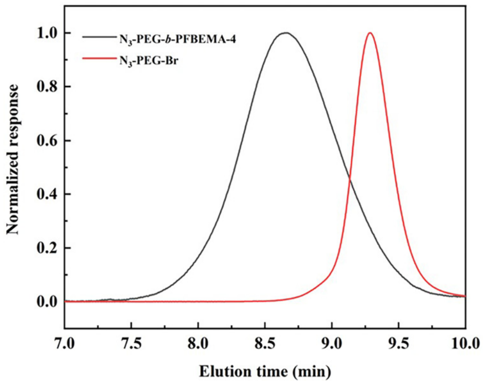

Figure S2. GPC curves of polymers (sample: N_3-PEG-*b*-PFBEMA-4, eluent: THF).

Table S1. Summary of molecular weight information of polymers synthesized with different feeding ratios.

Samples	PEG : PFBEMA (feed ratio m_1 : m_2)	$\overline{M}_n$ (g mol^{-1})[a]	$\overline{M}_w$ (g mol^{-1})[a]	$Đ$ [a]
N_3-PEG-*b*-PFBEMA-1	1.0：0.6	8700	10800	1.25
N_3-PEG-*b*-PFBEMA-2	1.0：0.8	11100	14000	1.27
N_3-PEG-*b*-PFBEMA-3	1.0：1.0	13900	19200	1.38
N_3-PEG-*b*-PFBEMA-4	1.0：1.2	15300	19100	1.25

[a] Determined by GPC with THF as the eluent and polystyrene as standards.

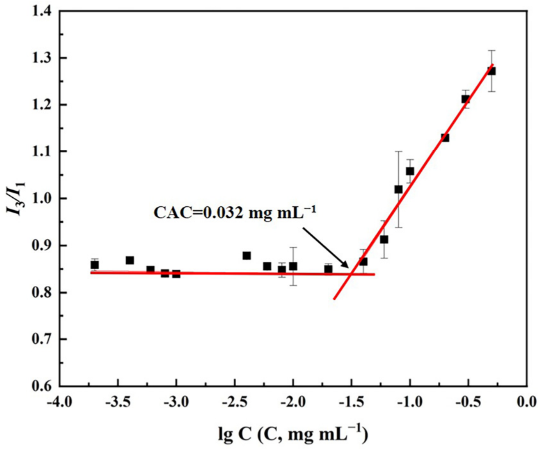

Figure S3. Curve of the relationship between fluorescence intensity ratio (I_3/I_1) and N_3-PEG-*b*-(PFBEMA-DOX) concentration (lg C) in pyrene fluorescence emission spectrum.

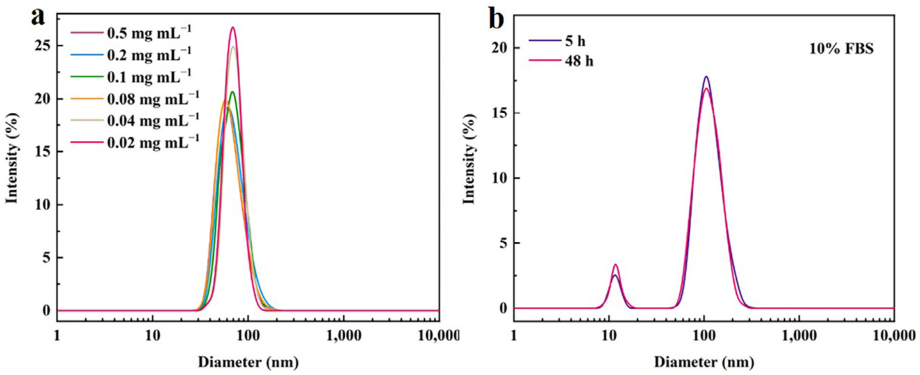

Figure S4. (a) The size distribution of mAb-CD163-PDNPs at different concentrations in PB 7.4 and (b) The size distribution of mAb-CD163-PDNPs in PB 7.4 containing 10% fetal bovine serum (FBS) stirred for 5 h and 48 h (Concentration: 0.5 mg mL^{-1}).

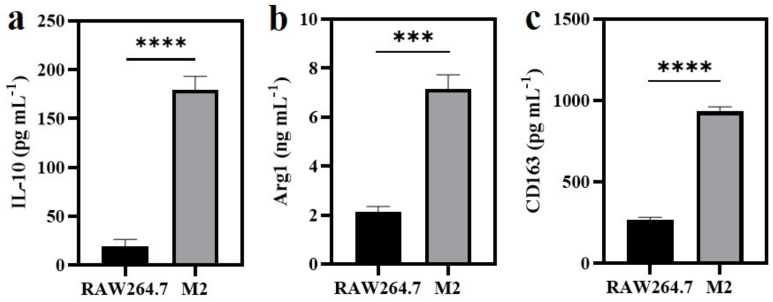

Figure S5. Concentration of (a) IL-10, (b) Arg1 and (c) CD163 in cell culture supernatant before and after RAW264.7 induction.
